# Nonresponse Error in Mail Surveys: Top Ten Problems

**DOI:** 10.1155/2011/987924

**Published:** 2011-05-05

**Authors:** Jeanette M. Daly, Julie K. Jones, Patricia L. Gereau, Barcey T. Levy

**Affiliations:** ^1^Department of Family Medicine, University of Iowa, 01290-F PFP, 200 Hawkins Drive, Iowa City, IA 52242, USA; ^2^Department of Integrative Physiology, University of Iowa, 424 Field House, Iowa City, IA 52242, USA

## Abstract

Conducting mail surveys can result in nonresponse error, which occurs when the potential participant is unwilling to participate or impossible to contact. Nonresponse can result in a reduction in precision of the study and may bias results. The purpose of this paper is to describe and make readers aware of a top ten list of mailed survey problems affecting the response rate encountered over time with different research projects, while utilizing the Dillman Total Design Method. Ten nonresponse error problems were identified, such as inserter machine gets sequence out of order, capitalization in databases, and mailing discarded by postal service. These ten mishaps can potentiate nonresponse errors, but there are ways to minimize their frequency. Suggestions offered stem from our own experiences during research projects. Our goal is to increase researchers' knowledge of nonresponse error problems and to offer solutions which can decrease nonresponse error in future projects.

## 1. Introduction

Conducting mail surveys can result in four potential sources of error: sampling error, noncoverage error, nonresponse error, and measurement error [1]. Nonresponse error occurs when the potential participant is unwilling to participate or impossible to contact. Nonresponse error can be divided into two categories: noncontacts and refusals [2]. 

Reasons for noncontact (when mailed surveys *do not reach* the potential subjects) could include the following: (a) insufficient postage, (b) incorrect mailing address, (c) bulk mail delay or nondelivery by post office, or (d) interception and disposal of mail by a family member or significant other. Reasons for refusals (when surveys may have reached the potential subject, but were not returned) could include the following: (a) no return address provided, (b) no postage-paid return envelope provided, (c) unclear survey instructions, (d) survey too long or complicated, (e) mistrust of confidentiality assurances, (f) insufficient incentive/payment, (g) unappealing survey topic, (h) lack of interest, or (i) competition with other mailings (just more junk mail).

Unfortunately, no matter how carefully a sample is selected, some potential participants do not respond. When those who respond to the mail survey differ by demographic characteristics from those who don't, nonresponse error can reduce the precision of the study and may bias results.

Researchers are aware of nonresponse error and have attempted to develop methods to alleviate this problem and obtain high response rates. One technique is the Total Design Method [3]. According to the theoretical framework of this method, questionnaire development and survey implementation methodology have three considerations: (1) reducing the perceived cost, (e.g., making the questionnaire short and easy to complete), (2) increasing perceived rewards, (e.g., making the questionnaire itself interesting to fill out), and (3) increasing trust (e.g., using official stationery and sponsorship). 

However, implementing these theoretical strategies in a mailed survey does not eliminate nonresponse error. Other unfortunate mishaps can affect the response rate. The following is a list of top ten mailed survey problems affecting the response rate that we have encountered over time with different research projects, while utilizing the Dillman Total Design Method [3].

**(10) Social Title Salutation Incorrect**

For customized salutations and social titles, the gender of the mail recipient needs to be accurate. In an electronic medical record data pull of 14000 names and addresses for one of our studies, the data included the person's name and gender. For some individuals, the gender was listed incorrectly, (e.g., John was labeled a female and Mary was labeled a male). Social and professional titles, such as Dr., Ms., or Mr., are important in the salutation and should be accurate. A recipient may be inclined to discard an item showing an incorrect title.

**(9) Remailing Expense Not Included in Budget**

All research entails budgeting for expenses. Budgeting for a mailing can include recruitment costs, subject incentives, paper, envelopes, copying, and postage for multiple mailings that include a preletter, baseline cover letter and survey, reminder letter, and follow-up cover letter and survey. This can be expensive. The amount budgeted is based on the proposed sample size; and, inversely, the sample size could at times be constrained by the cost of the mailing. When part of a mailing has been discarded by the post office (see (1) below), there may not be enough funding for a remailing. This forces a nonresponse error that impacts the return rate and power of the study.

**(8) Human Error While Stuffing Envelopes**

In survey mailings processed by one or more persons, human error can occur: an envelope may be missing an item such as the incentive, questionnaire, postage-paid return envelope, or cover letter. Another mishap that can occur is a mismatch between the cover letter and identification number for the questionnaire. Most survey mailings include a return addressed envelope that may or may not be postage-paid. If a return address is not enclosed by any mechanism in the package, such as a postage-paid return envelope, there will be no way for the receiver to return the questionnaire.

**(7) Letter Folder/Inserter Machines Get Sequence out of Order**

Just as humans can get a sorting sequence out of order, electronic folder and inserter machines can do the same. This may result in a mismatch between the address on an outside envelope, its enclosed cover letter, and/or the return envelope.

**(6) Incorrect Address Is Used**

The name and address list obtained for a mailing may have incorrect addresses, causing letters to be returned to the sender or discarded.

**(5) Commercial Pricing = Junk Mail Look Alike**

**Figure 1:**
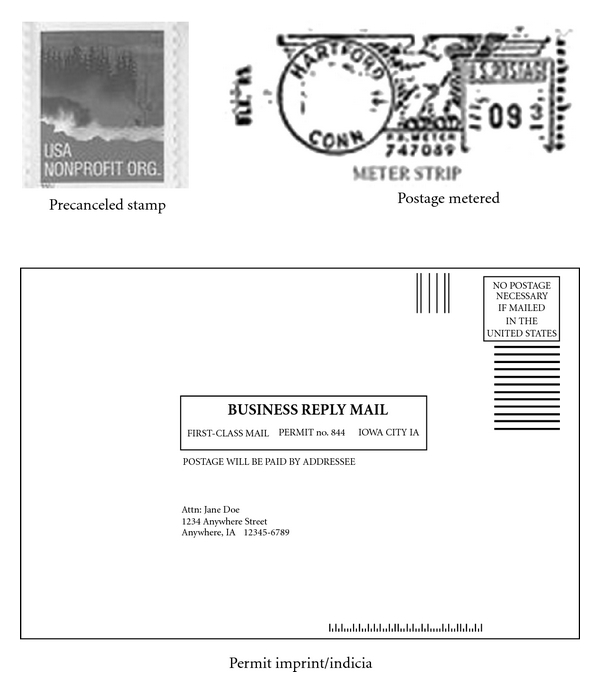
Depiction of precanceled stamp, postage metered, and permit imprint.

Commercial pricing or bulk mail rates are available for use with precanceled stamps, metered postage, or a permit imprint (see Figure 1). Recipients may be more likely to discard such mailings without opening them, assuming them to be “junk” mail.

**(4) Capitalization of Address Databases**

The names and addresses in many database files from electronic medical records are in all-capital letters. Although Microsoft Excel has a function to automatically change the first letter to uppercase and the remaining letters to lowercase, the results with certain names, such as McKinley reading Mckinley or 1st Street reading 1st Street, can cause potential mailing and delivery errors.

**(3) Incorrect Mailing List Is Provided**

In most cases, to generate a mailing list, a series of inclusion and exclusion criteria is specified. Administrators of an agency are involved with the decision and protocol to release the information. In one instance, we had requested a list of patients meeting certain inclusion criteria from a primary care office. After receiving the list, we implemented the Dillman Method [3]; and, during the reminder follow-up telephone calls, several subjects told us they had never been patients at that primary care office. We then contacted the administrator and learned that the list had been pulled from the entire hospital electronic data base, as the office did not have the electronic capability to generate a list which included only their clinic's patients. They had not reviewed the list to determine which persons attended their clinic. This was an unusual occurrence, and a reportable event form was completed for the institutional review board.

**(2) Deceased Person Is Sent a Mailing**

With electronic medical record data pulls, a potential subject list can include the names of deceased persons. The criteria for the data pull should explicitly exclude those persons. Unfortunately, the data set is only as good as the person keeping it current. If deaths are not immediately recorded in the medical record, the information will not be up to date.

**(1) Mailing Is Discarded by Post Office (Sometimes Containing Monetary Incentive)**

Bulk mail can be discarded at the discretion of the postmaster if the addressee has a post office box number but the street address is used instead. Unfortunately, we learned of this through a mailing to over 500 subjects that included a $2 bill. The postmaster discarded approximately 60 envelopes mailed to post office box customers, because only the street address was used. Serendipitously, a postal worker received one of the mailings at home and discovered the $2 enclosure. During later follow-up telephone calls, we identified several persons who did not receive the mailing.

## 2. Suggested Solutions for Nonresponse Error Mishaps

Although these ten mishaps can potentiate nonresponse errors, there are ways to minimize their frequency. Many of the following suggestions are applicable to the various problems described above and are aimed at reducing the nonresponse rate. Research has been conducted on the survey process to determine best practices for achieving good response rates. The variables considered included length and content of the questionnaire, incentives, number of contacts, appearance, and the delivery process [4]. Suggestions offered stem from our own trial and error during research project mailings while using the Dillman Total Design Method [3]. The goal of this writing is to increase researchers' knowledge of nonresponse error problems and to offer solutions which can decrease nonresponse error in future projects.

## 3. Bulk Mailing

Bulk mailing is used for large quantities of mail, which can be processed at reduced postage rates. The mail is presorted in a zip code order which varies, depending on the size of the envelope. A mailing must contain a minimum of 200 pieces to qualify for a bulk mail postage discount. If a mailing is not quite 200 pieces, the names and addresses of research team members can be added to the list to check the success of the mailing process, which provides an additional benefit: the researcher will find out whether or not the bulk mailing was sent and, if sent, how long it took for potential subjects to receive the mailing. Sending the minimum quantity can generate considerable savings in postal expenses. For example, 200 #10 envelopes, weighing less than one ounce apiece would cost $88.00 to send via first-class mail. Using bulk mail, this same mailing would cost $27.09.

Franked envelopes, those marked to indicate the postage is paid, can be used on both the external (outgoing) envelope and the postage-paid return envelope. Several studies have been done to see if the survey response rate is negatively impacted by using franked envelopes as opposed to postage stamps. In one study, using an estimated pooled odds ratio with a random-effects model for six trials (*N* = 13,964), there was no evidence found that in outgoing mail with stamps the response was superior to that of franked envelopes (OR 0.95; 95% CI 0.88 to 1.03) [4]. However, in 27 other trials (*N* = 48,612) of enclosed postage-paid return envelopes, the odds of response was increased by 25% when stamps were used compared to franking (OR 1.24; 95% CI 1.14 to 1.35) [4]. Using stamps on return envelopes will increase the cost because there is no postage charged for franked envelopes unless they are returned. However, using franked envelopes may generate a lower response rate.

## 4. Human or Machine Error

Human error is likely to occur with each mailing. To account for the possibility of mismatching cover letter and identification coded material, envelopes should not be sealed until all have been stuffed. Randomly checking about every tenth envelope for accuracy is also a good practice.

Variable-data printing (VDP) is an efficient and economic method of on-demand printing in which elements of the text are changed from one printed piece to the next. This is also known as mail-merge printing. Printing fields are supplied by a database allowing for mass customization of documents via digital print technology, rather than using mass production of a single document. This process is increasingly integrating mailing with printing. In addition, a letter can now be folded, inserted into an envelope, and sealed by machine. However, letter folding/inserting machines do have limitations. Persons who have worked with the machines should have a manual process to check every 10th envelope to verify that the letter and envelope addresses match. The USPS web site (http://www.usps.com/) has detailed information about business mail.

## 5. Mailing Name and Address List

Each year about forty million Americans move either their business or place of residence. Unfortunately, their old addresses remain in many databases, so approximately 8% of all mail is undeliverable [5]. Researchers obtain names and addresses from a variety of sources. Whether from electronic data pulls or lists purchased from companies, addresses can change quickly. The U.S. Postal Service offers services to improve address reliability and quality, which help eliminate undeliverable mail and ensure that it reaches the intended subject. The U.S. Postal Service now requires bulk mailers to update their mailing lists every 90 days using an approved system. All presorted or automated mailings must comply in order to receive bulk mail postage discounts. A software program matches addresses to the U.S. Postal Service's National Database of known addresses, thus cleansing the address lists.

The address list cleansing is completed by a U.S. Postal Service-approved process such as National Change of Address (NCOA), NCOA Link, or FastForward and CodingAccuracy Support System (CASS) postal approved software. These programs can be costly and time consuming and must be regularly maintained with updates and changes. However, the cost is still substantially less than full first-class postage rates. 

Researchers working with agencies or institutions to obtain mailing lists should discover the source of the information being provided. After selecting subjects from a lengthy mailing list, a few employees from the institution could manually review the list to remove the names of persons they know are deceased. In addition, the gender of the potential subjects has to be confirmed.

## 6. Discarded Mail

Every year the Post Office discards approximately 35% of all bulk mail because it cannot be delivered to the addressee [6]. Prior to conducting a large mailing, a researcher should contact the Postal Service to discuss USPS policy and procedures for delivering bulk mail. Bulk mail can be discarded if the address is incorrectly written, ambiguous, or there is no such address known. It can also be discarded if the address is legitimate but the recipient is no longer at that location.

Mailing list cleanup can help alleviate this problem. post office box numbers should be used if available, rather than street addresses. Inform the postmaster of the content of the bulk mailing. Be aware that if incentives such as gift cards or money are in the mailing, they can be unknowingly discarded along with the mailing.

## 7. Institutional Review Board Reportable Events

Mailing mishaps that occur may be reportable events to the institutional review board (IRB). Investigators must report any unanticipated problem involving risk to subjects or others using a Reportable Event Form. This form includes a description of the event, the date of occurrence, whether it is a local or outside report, how the event affected the rights, safety or welfare of the subject or others, current status of subjects, and any planned changes or modifications to the project as a result of the event. Although the reporting is mandatory and valuable, it forces the researcher to use his or her time filing reports rather than doing research. 

## 8. Conclusion

Nonresponse survey error is typically thought of as mail that has undeliverable address or a lack of interest in participation by the potential subject, but many other factors can affect the nonresponse error rate. A great number of these can be remedied with a few extra steps, such as mail cleansing, double-checking stuffed envelopes, informing the postmaster ahead of time about a particular mailing, and working closely in great detail with the person providing the mail list.
